# Comparative Analysis of αB-Crystallin Expression in Heat-Stressed Myocardial Cells *In Vivo* and *In Vitro*

**DOI:** 10.1371/journal.pone.0086937

**Published:** 2014-01-22

**Authors:** Shu Tang, Yingjun Lv, Hongbo Chen, Abdelnasir Adam, Yanfen Cheng, Jörg Hartung, Endong Bao

**Affiliations:** 1 College of Veterinary Medicine, Nanjing Agricultural University, Nanjing, China; 2 Institute for Animal Hygiene, Animal Welfare and Farm Animal Behaviour, University of Veterinary Medicine Hannover, Foundation, Hannover, Germany; Case Western Reserve University, United States of America

## Abstract

Relationships between αB-crystallin expression patterns and pathological changes of myocardial cells after heat stress were examined *in vitro* and *in vivo* in this study using the H_9_C_2_ cell line and Sprague-Dawley rats, respectively. Histopathological lesions, characterized by acute degeneration, karyopyknosis and loss of a defined nucleus, became more severe in rat hearts over the course of heat stress treatment from 20 min to 100 min. The expression of αB-crystallin in rat hearts showed a significant decrease (*P*<0.05) throughout the heat stress treatment period, except at the 40 min time point. Likewise, decreased αB-crystallin expression was also observed in the H_9_C_2_ cell line exposed to a high temperature *in vitro*, although its expression recovered to normal levels at later time points (80 and 100 min) and the cellular damage was less severe. The results suggest that αB-crystallin is mobilized early after exposure to a high temperature to interact with damaged proteins but that the myocardial cells cannot produce sufficient αB-crystallin for protection against heat stress. Lower αB-crystallin expression levels were accompanied by obvious cell/tissue damage, suggesting that the abundance of this protein is associated with protective effects in myocardial cells *in vitro* and *in vivo*. Thus, αB-crystallin is a potential biomarker of heat stress.

## Introduction

Heat is a naturally occurring factor affecting mammalian reproduction. However, uncontrolled heat can lead to serious consequences, and a body temperature above 42±1°C generally is considered to be life-threatening [1]. Some studies have confirmed that this high body temperature is associated with heat stroke onset, when the disruption in homeostasis results in thrombocytopenia, disseminated intravascular coagulation (DIC) [2], [3] or even sudden death [4], [5]. In the period from 1979 to 1997, approximately 7,000 deaths were attributed to excessive heat in the United States [6]. Similar incidences of mortality associated with high temperature have been reported in other studies in the United States and warm climate countries such as Saudi Arabia [7], [8]. The cause of the progression from heat stress to lethal heat stroke is currently unknown, but some researchers have suggested that the mechanism of hyperthermia-induced sudden death involves an increase in metabolic demand, resulting in heart failure, heart attack, stroke, cardiac arrest [1], [9] and arterial hypotension [10]. The heart is a vital organ with a high metabolic demand. Cardiomyocytes are engaged continuously in generating the necessary contractile force to sustain cardiac output to the circulation throughout the body [11], and a higher body temperature can lead to disruption of function, proliferation and structure of these cells [12]. High heat stress-induced mortality also has been documented in animals, such as the nearly 40% death rate in broiler chickens exposed to the temperature of 40°C [13].

The heat shock response (HSR) is an evolutionarily conserved defense mechanism against sudden stresses, such as elevated temperatures or environmental changes. Heat shock proteins (HSPs) have been detected *in vivo* and *in vitro* at all temperatures [14]–[16], but they are induced to a higher level when subjected to heat. Most HSPs are molecular chaperones that play important roles in repair and removal of misfolded and denatured proteins, thereby conserving cellular protein homeostasis [17]–[19]. The Hsp70 family has been extensively researched, and high preoperative levels of its members can reduce the deleterious effect of ischemia–reperfusion *in vivo* [20]–[23]. HSPs fall into six families according to their molecular weight: HSP110, HSP90, HSP70, HSP60, HSP47 and small heat shock protein (sHSP) families [18]. Previous investigations have detected sHSPs in the heart and distributed in the cytosol of cardiac cells [24]–[27], especially αB-crystallin, which is one of the true sHSPs that display enhanced synthesis in response to several stresses in humans [28]–[30].

αB-crystallin can be found in most organisms to respond to several unfavorable stresses (e.g., UV, hyperthermia, toxic radicals) to protect cells [31]–[33]. The mechanism of this protection involves the organization of cytoskeletal structures, prevention of the accumulation of denatured proteins and increase of cellular tolerance to stress [34]. αB-crystallin is widely expressed in the heart, skeletal muscle and other organs in the absence of stress *in vivo* [35]. Furthermore, αB-crystallin has been localized to the I-band and M-line region of myofibrils and confirmed to have a myofibril-stabilizing role in cardiomyocytes *in vitro* [36]. αB-crystallin also promotes cell survival and inhibits apoptosis following its induction by cellular stresses, including heat and reactive oxygen species [37]. The induction of αB-crystallin in the myocardium has been suggested to be a cardioprotective cellular response [38]. However, the relationships between pathological changes of heart cells/tissues and expression of αB-crystallin in response to heat stress *in vitro* and *in vivo* are not fully understood; thus, they were investigated in this study, respectively, by exposure of the whole rat body and a myocardial cell line to high temperature. The findings provide implications for the role of αB-crystallin in the protection of cardiac cells against heat stress.

## Materials and Methods

### Animals and Experimental Design

All experiments were performed in accordance with the guidelines of the Animal Ethics Committee of Jiangsu Province (China) and were approved by the Institutional Animal Care and Use Committee of Nanjing Agricultural University, China. Sixty-day-old Sprague-Dawley (SD) rats (n = 60) were purchased from the Qinglongshan Farm (Nanjing, China) and maintained at room temperature (RT) at 25°C for 5 days. Thereafter, the rats were randomly divided into six groups (n = 10 per group) and subjected to different periods of heat stress (control, 20 min, 40 min, 60 min, 80 min, 100 min). While the control rats were kept at RT, animals of the other five groups were immediately transferred into a controlled climate chamber (New Jiangnan Instrument Co., Ltd; Ningbo, Zhejiang) pre-heated to 42°C, with certified fresh air and relative humidity between 55–65%. During the course of heat stress treatment, water and food were supplied, and the mental state and activities of rats were observed and noted. Within 3 min of the end of the designated heat stress period, blood was collected from the rats, which were sacrificed immediately thereafter. Heart samples were collected and fixed in formalin for pathological observation or stored in liquid nitrogen for Western blot analysis.

### Cell Culture and Preparation

The H_9_C_2_ myocardial cell line was purchased from American Type Culture Collection (ATCC, Shanghai, China) and cultured in Dulbecco’s modified Eagle’s medium (DMEM), supplemented with 10% fetal calf serum (FBS) in an incubator at 37°C, until the confluency was greater than 90%. Cells were divided into six groups for exposure to different periods of heat stress. Except for the control group kept in a 37°C incubator with a humidified atmosphere of 5% CO_2_ and 95% air, the other five groups were exposed to heat stress for 20, 40, 60, 80 or 100 min. Heat stress treatment was achieved as quickly as possible by changing the temperature in the incubator from 37°C to 42°C with a humidified atmosphere of 5% CO_2_ and 95% air.

### Histo- and Cytopathological Examination

Heart samples were fixed in formalin, cut into 4-µm serial sections after embedding in paraffin and stained with hematoxylin and eosin (H&E). Heat-stressed H_9_C_2_ cells (2–8×10^4^ cells in 35 mm^2^ plates) grown on glass coverslips coated with poly-L-lysine were washed with PBS three times after discarding the medium and then fixed in 95% alcohol for 20 min. Thereafter, the cells were washed with PBS three times (∼1 min each time) and stained with hematoxylin for 1 min. After washing with tap water for 5 min, the coverslips were dipped in acid alcohol and then rinsed again with tap water before staining with eosin for 1 min. After being dehydrated in accending concentrations of alcohol (75%, 95% and 100%) for 1–2 min each and cleared two times with xylene for 5 min each, the coverslips were mounted on slides using a mounting agent and observed under a light microscope (Axio Imager A2, Zeiss, Jena, Germany).

### Immunofluorescent Staining

Dewaxed heart tissue sections (4 µm) were fixed with hydrochloric acid (HCl) solution for antigen retrieval (2 N HCL in distilled water, pH 0.6–0.9) for 20 min at RT. After washing with PBS three times, endogenous peroxidase activity was inactivated by incubation in 3% (v/v) H_2_O_2_ for 10 min at RT. Subsequently, the sections were blocked with 5% bovine serum albumin (BSA) for 30 min at 37°C and then incubated with the αB-crystallin primary antibody (ADI-SPA-222-F, Enzo Life Science, USA) at 1∶100 dilution for 2 h at 37°C. The negative controls were coated with 1% BSA. After washing with PBS containing 1% Tween-20 three times, sections were incubated with a horseradish peroxidase goat anti-mouse IgG-HRP (H+L) secondary antibody at 1∶500 dilution for 1 h at 37°C. The sections were washed with PBS containing 1% Tween-20 three times and then treated with two drops of ready-made 3, 3′-diaminobenzidine (DAB) substrate chromogen solution for 15 min until the desired brown color appeared. The sections were counterstained with hematoxylin and observed under a light microscope (Axio Imager A2, Zeiss, Jena, Germany).

H_9_C_2_ cells (2–8×10^4^ cells in 35 mm^2^ plates) were fixed directly on the plates using pre-cooled 3% formaldehyde in PBS for 30 min at RT and permeabilized with 0.1% Triton X-100 in PBS for 10 min. After blocking with 5% skim milk in PBS for 1 h, a 1∶200 dilution of the αB-crystallin monoclonal antibody was added to the coverslips and incubated in a moist chamber for 1 h at 37°C. After washing in PBS three times, the coverslips were incubated with a FITC-conjugated goat anti-mouse IgG antibody at a 1∶500 dilution (BA1101, Boster, Wuhan, China) at 37°C for 1 h. After washing again with PBS, the coverslips were stained with DAPI solution (H-1000, Vector Laboratories, Burlingame, CA, USA) and observed using an immunofluorescence microscope (Axio Imager A2, Zeiss).

### Western Blotting

After 0, 20, 40, 60, 80 and 100 min of heat stress treatment in an incubator at 42°C, H_9_C_2_ cells were washed two times with PBS and lysed in M-PER® mammalian protein extraction reagent (28501, Thermo Scientific, Waltham, MA, USA) supplemented with Halt™ protease inhibitor cocktail according to the manufacturer’s instructions. The cell homogenates were then centrifuged at 14,000×*g* for 5 min at 4°C, and the supernatants were used as total protein extracts.

All experimental rats were humanely sacrificed by decapitation. Approximately 100 µg of heart tissue was taken for each specimen, and 1 mL of PBS was added for homogenization using a Fluko® Super Fine Homogenizer (623003, Fluko Equipment Shanghai Co. Ltd, Shanghai, China), followed by centrifugation at 2,000 rpm for 15 min. The cell pellets were resuspended in 200 µl of ice-cold RIPA lysis buffer (50 mM Tris, pH 7.4, 150 mM NaCl, 1% NP-40, 0.5% sodium deoxycholate, 0.1% SDS and l ml phenylmethanesulfonyl fluoride (PMSF) (WB-0071, Beijing Dingguo Changsheng Biotechnology Co. Ltd, Beijing, China).

The protein content was measured using the BCA protein assay kit (Thermo Scientific) as previously described [39], [40] with an ELISA plate reader (Argus 300, Packard, St Cyr, France). H_9_C_2_ cell (20 µg) and heart (20 µg) sample proteins were loaded on a 13% acrylamide gel with a 4% stacking acrylamide gel and migrated by electrophoresis in a buffer containing 25 mM Tris, pH 7.6, 0.1% SDS and 0.2 M glycine. After separation, the proteins were transferred onto Hybond C membranes (Amersham Bioscience, Little Chalfont, Bucks, UK) for 75 min using a buffer containing 25 mM Tris base, pH 7.6, 0, 1% SDS, 0.2 M glycine and 20% methanol. Blots were rinsed four times in wash buffer [20 mM Tris base, pH 7.6, 12.5 mM NaCl and 0.05% Tween-20 (TBST)] and blocked for 1 h at RT in TBST buffer containing 5% milk powder at RT. Subsequently, the membranes were incubated overnight at RT with the primary aB-crystallin mouse monoclonal IgG antibody, washed 3×5 min in TBST buffer and incubated with TBST buffer containing 5% skim milk powder and the secondary goat anti-mouse IgG antibody (SN133, Sunshine Biotech, Nanjing Co. Ltd. Nanjing, China). After washing 3×5 min in TBST buffer again, bands were revealed using DAB substrate (Sigma, St. Louis, MO, USA) in 30 mL of Tris buffer (60 mM, pH 6.8) containing 0.2% H_2_O_2_ and 200 µL of 0.8% NiCI_2_. After staining, the membranes were washed in distilled water and dried. The bands on the developed film were quantified with Quantity One 4.6.2 software (Bio-Rad, Hercules, CA, USA). The density of each band was normalized to that of the GADPH protein.

### Statistical Analysis

Differences between the heat stress groups and the control group were analyzed by one-way analysis of variance (ANOVA), followed by the least significant difference (LSD) multiple comparison test, using the Statistical Package for Social Sciences (SPSS version 20.0 for Windows). Results were expressed as the mean ± standard deviation (SD) of at least three independent experiments. *P* values <0.05 (*) or <0.01 (**) were considered statistically significant. All experiments were performed in triplicate (n = 3).

## Results

### Clinical Symptoms of Heat-stressed Rats

As soon as they were moved into the pre-heated chamber at 42°C from an environment at 25°C, all rats began to show polypnea and sensitivity to heat stress compared with the control group. After 20 min of heat stress, drinking behavior of the rats appeared to increase. The first rat death induced by heat occurred after 45 min of heat stress. At 60 min of heat stress, 5% of the rats were dead. With increased duration of heat stress to 80 min, the death rate increased to 50% of the rats. At 100 min and 120 min of heat stress, 50% and 100% mortality rates were recorded, respectively.

### Histo- and Cytopathological Lesions of Heat-stressed Rat Hearts

**Figure 1:**
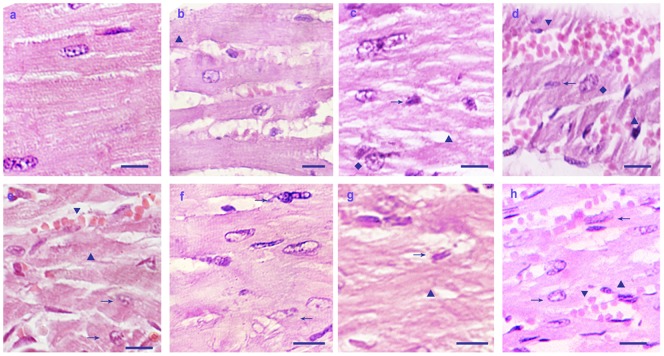
Histopathological lesions of rat hearts heat-stressed *in vivo*. H&E staining, scale bar = 10 µm. (a) No obvious pathological changes were observed in control rats. In heat-stressed rat hearts, the following were observed at the indicated time points: (b) 20 min, acute granular degeneration (▴) with enlargement of heart cells(♦); (c) 40 min, swollen myocardial cells with reduced nuclei and cloudy cytoplasm (→♦); (d) 60 min, enlarged myocardial cells with acute granular degeneration (▴), accompanied by bleeding (▾); (e) 80 min, myocardial cells with cloudy cytoplasm and karyopyknosis (→); (f) 100 min, loss of defined nuclei (←) in enlarged myocardial cells. (g) In hearts of dead rats at 80 min of heat stress, pathological lesions, characterized by disordered arrangement of cells and loss of striations, along with karyopyknosis (→) and loss of nuclear definition (←), were observed. (h) In hearts of dead rats at 100 min of heat stress, pathological lesions were observed, characterized by karyopyknosis (→) and granular degeneration (▴), accompanied by bleeding (▾).

Histopathological changes of rat hearts heat-treated *in vivo* are shown in Figure 1 (a–f). Unlike the control group, acute degenerative lesions in the rat hearts were seen at the beginning of the heat stress treatment (from 20 min), characterized by a higher density of fine cytoplasmic granules in the cytoplasm (Fig. 1b–f▴). At 20 and 40 min of heat stress treatment, the granular degeneration of the myocardial cells was recognized by light pink staining, tiny granular particles and loss of striations in the cytoplasm (Fig. 1b–f▴). The nuclei of myocardial cells were swollen, some of which were nearly disintegrated, and the space between muscle fibers widened (Fig. 1b–e♦). After 60 min of exposure to high temperature, hyperemia was observed due to the increased capillary blood flow produced by arteriolar dilation (Fig. 1e▾). The most severely damaged myocardial cells appeared to have lost a defined shape and were in a state of karyopyknosis (Fig. 1c–f→) with most of the nuclei shrunken or nearly disappeared (Fig. 1c–f←). By comparison, no obvious pathological changes were observed in control rats.

Histopathological lesions of heart samples from rats that died of heat stress are shown in Figure 1g and h. Slight granular degeneration of cardiac muscle cells was observed in the heart sections of all rats with heat-induced death(Fig. 1g and h▴), whereas no marked lesions were detected in control rats. The myocardial cells showed acute granular degeneration characterized by light pink staining, small granular particles and loss of striations in the cytoplasm (Fig. 1h▴). The severely damaged myocardial cells lost their defined shape, and most of the cells showed marked basophilic karyopyknosis and nuclear disintegration (Fig. 1g and h→). Bleeding was also observed in the heart tissue, accompanied by obvious hyperemia (Fig. 1h▾).

**Figure 2:**
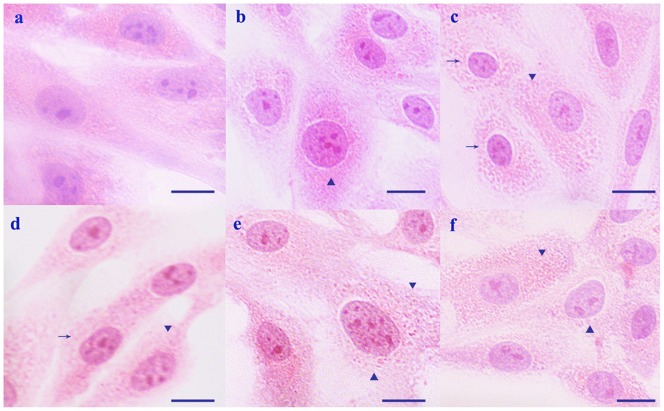
Cytopathological lesions of H_9_C_2_ cells heat-stressed *in vitro*. H&E staining, scale bar = 10 µm. (a) No obvious pathological changes were observed in control H_9_C_2_ cells. In heat-stressed H_9_C_2_ cells, the following were observed at the indicated time points: (b) 20 min, acute degeneration characterized by enlarged cell size (▴); (c) 40 min, light and clear pink granulation (▾) in the cytoplasm and karyopyknosis (→) was observed; (d) 60 min, cloudy cytoplasm (▴). (e) 80 min, enlarged cell size (▴) and intracellular granules (▾); (f) 100 min, acute granular degeneration characterized by numerous pink granules (▾) and enlarged cell size (▴).

Cytopathological changes of the H_9_C_2_ rat myocardial cell line heat-treated *in vitro* are shown in Figure 2. Acute degeneration characterized by enlarged cellular size (Fig. 2b–f▴) and pink granules (Fig. 2b–f▾) were observed throughout the course of heat stress treatment (from 20 min to 100 min). At 40 and 60 min of heat stress, necrosis characterized by karyopyknosis and loss of nuclei were observed (Fig. 2c and d→). At 80 min and 100 min of heat stress, obvious pink granules were observed in the enlarged stress-damaged myocardial cells (Fig. 2e–f▴). No obvious pathological changes were seen in the control myocardial cells.

### Localization of αB-crystallin in Heart Tissue and H_9_C_2_ Cells

**Figure 3:**
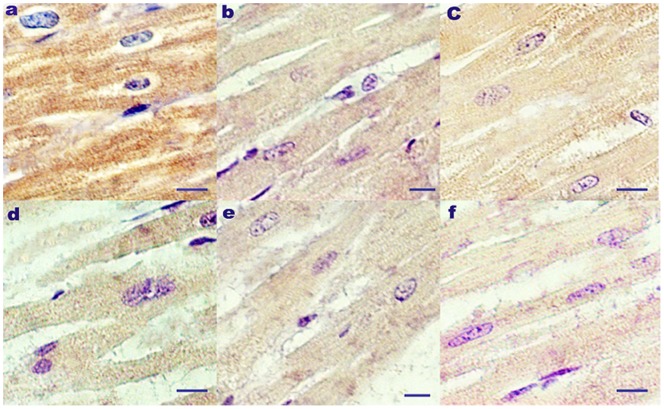
Localization of αB-crystallin in rat heart tissue before and after heat stress *in vivo*. Immunohistochemical staining, scale bar = 10 µm, αB-crystallin (brown color, DAB). (a) Positive αB-crystallin signals were localized in the cytoplasm of non-stressed rat heart cells. In heat-stressed rat hearts, the following were observed at the indicated time points: (b) 20 min, αB-crystallin signals were positive mainly in the cytoplasm but weaker compared to the control group; (c) 40 min, αB-crystallin signals were still detected in the cytoplasm but stronger than at 20 min of heat stress; (d) 60 min, αB-crystallin was still localized in the cytoplasm; (e) 80 min, weaker positive αB-crystallin signals were still mainly expressed in the cytoplasm; (f) 100 min, αB-crystallin was expressed weakly in the cytoplasm.

**Figure 4:**
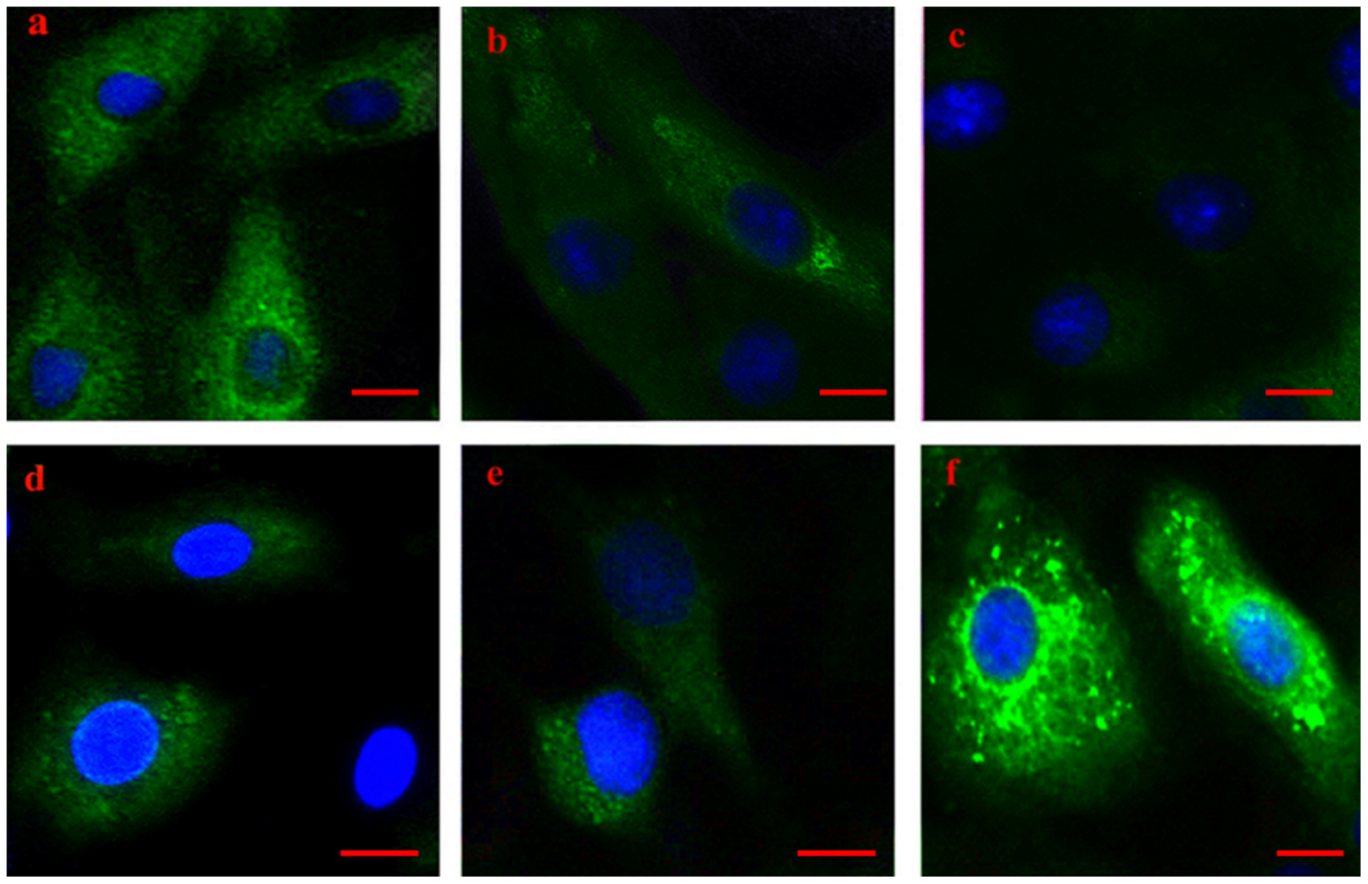
Localization of αB-crystallin in H_9_C_2_ cells before and after heat stress *in vitro*. Immunocytochemical staining, scale bar = 10 µm. αB-crystallin (green color, FITC), nuclei (blue color, DAPI) (a) αB-crystallin was localized in the cytoplasm of non-stressed H_9_C_2_ cells. In heat-stressed H_9_C_2_ cells, the following were observed at the indicated time points: (b) 20 min, weaker positive αB-crystallin signals localized in the cytoplasm, compared with those of control group; (c) 40 min, αB-crystallin signals localized weakly in the cytoplasm; (d) 60 min, αB-crystallin signals were persistently weaker both in the cytoplasm and over the nucleus; (e) 80 min, αB-crystallin still localized over the nucleus and in the cytoplasm at a lower level; (f) 100 min, most strongly positive αB-crystallin signals localized in the cytoplasm as well as around and over the nucleus.

Localization patterns of αB-crystallin in rat heart sections and cultured H_9_C_2_ cells are shown in Figures 3 and 4, respectively. In the rat heart, αB-crystallin was localized in the cytoplasm of cardiac cells both before and after heat stress. However, the densities of αB-crystallin in different heat-stressed groups varied. Near the beginning of the heat stress treatment (20 min), positive αB-crystallin signals in the cytoplasm of heart cells were detected, but the density was decreased compared to that of the control group. Except for its strong expression in the cytoplasm of myocardial cells at 40 min of heat stress, weaker αB-crystallin signals were observed in the cytoplasm after 60 min of heat stress compared to the earlier time points. After 80 min of heat stress, even weaker positive αB-crystallin signals were observed compared to those of the 60 min group. The weakest αB-crystallin signal was observed at 100 min of heat stress. In the control group, αB-crystallin was evenly distributed in the cytoplasm of heart cells at a relatively high density.

In the H_9_C_2_ myocardial cell line, αB-crystallin was mainly localized in the cytoplasm of the normal control group. Compared with the control group, changes in the pattern of αB-crystallin expression were apparent in the heat-stressed groups. At 20 min and especially 40 min of heat stress, positive but weak αB-crystallin signals were localized in the cytoplasm, compared with those of control group. At 60 and 80 min of heat stress, the αB-crystallin signals remained weaker in the cytoplasm but also appeared over the nucleus. At 100 min of heat stress, the strongest αB-crystallin signals were observed mainly as bright particles in the cytoplasm but also surrounding and overlaying the nuclear area of the myocardial cell. However, it could not be determined from the fluorescent images whether some of the αB-crystallin signals originated from the nucleus itself or solely from the cytoplasm above the nucleus. While the latter conclusion is likely the case, further studies are warranted to determine if αB-crystallin molecules are transferred to the nucleus under prolonged heat stress.

### Expression of αB-crystallin in Heat-stressed Rat Myocardial Cells *in vivo* and *in vitro*

**Figure 5:**
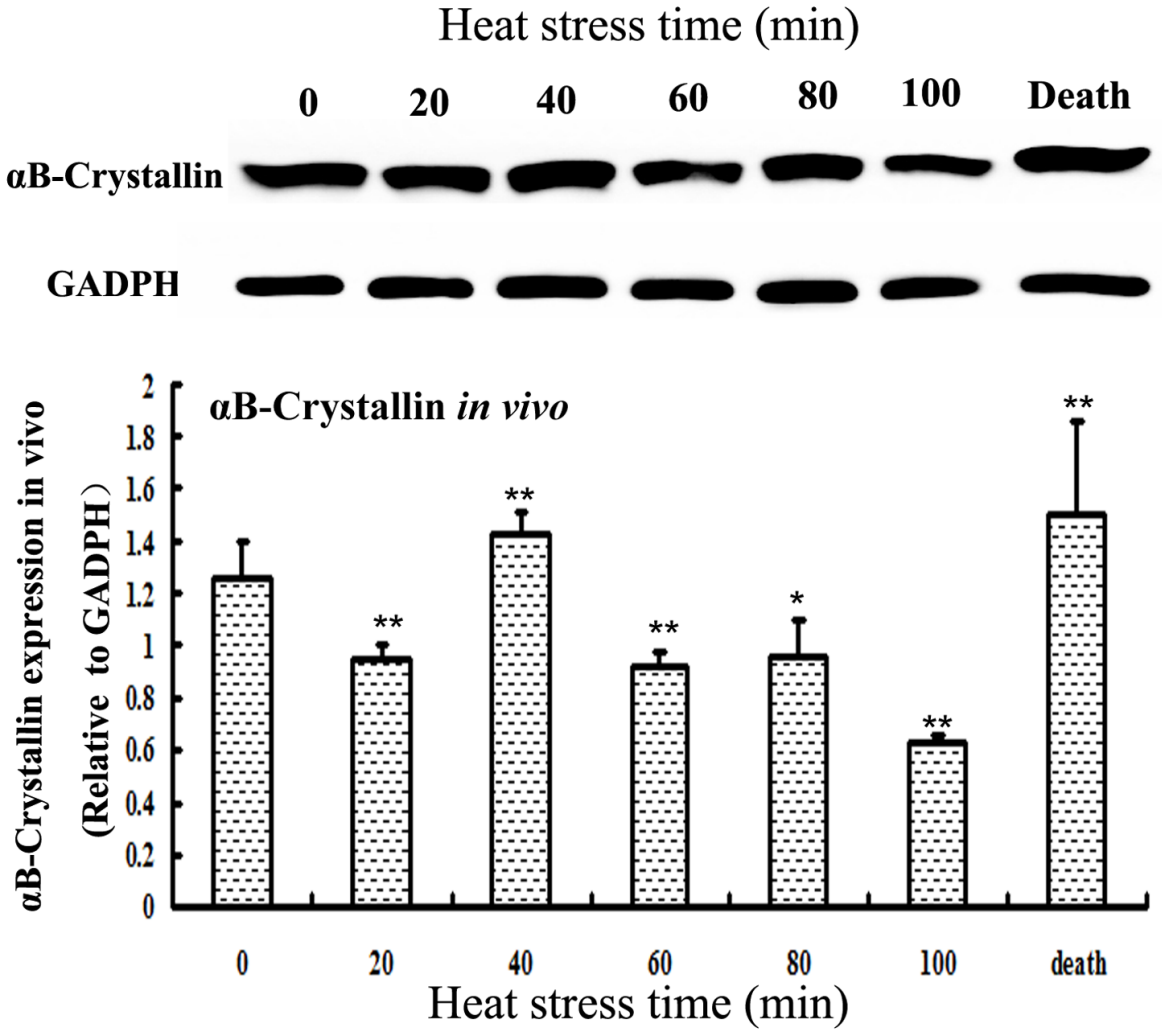
αB-crystallin expression in rat hearts heat-treated *in vivo*. αB-crystallin expression in rat heart tissue decreased (*P*<0.01) at 20 min of heat stress compared to the control and then significantly increased (*P*<0.01) at 40 min of heat stress. After 60 min of heat stress, αB-crystallin expression levels decreased again (*P*<0.01) and remained at lower levels until 100 min of heat stress. However, the level of αB-crystallin in dead rats was much higher than those of the control and all other heat-stressed groups. ** *P*<0.01; *P<0.05.

**Figure 6:**
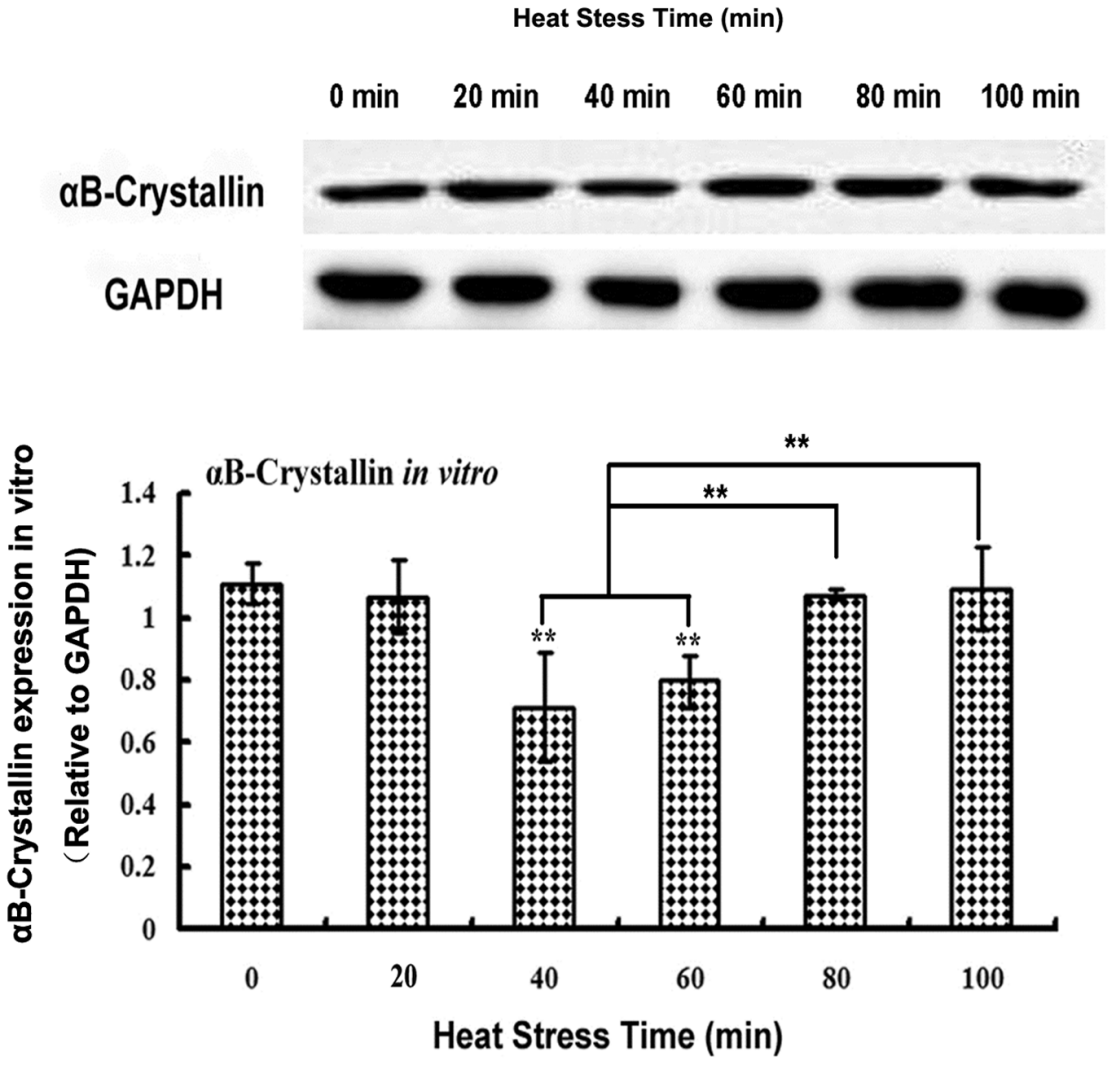
αB-crystallin expression in H_9_C_2_ cells heat treated *in vitro.* αB-crystallin decreased significantly (*P*<0.01) from 40 min to 60 min of heat stress compared to the control group. However, from 80 min to 100 min of heat stress, αB-crystallin levels increased and were not significantly different from that of the control group. ** *P*<0.01; *P<0.05.

Variations in expression of αB-crystallin protein in heat-stressed rat hearts and H_9_C_2_ cells, normalized to the corresponding GAPDH protein expression, are shown in Figures 5 and 6, respectively. Except for the 40 min heat stress group, αB-crystallin expression in the rat heart from the beginning of exposure to heat (20 min) until the end of the heat stress period (100 min) was decreased significantly (*P*<0.01) compared with the control group (0 min). Interestingly, levels of αB-crystallin expression in the hearts of rats that died between 40 min to 100 min of heat stress were significantly (*P*<0.01) higher than those of all heat-stressed rats and control rats.

In the H_9_C_2_ cell line, the level of αB-crystallin expression did not initially change at 20 min of heat stress but then decreased significantly (*P*<0.01) at the 40 min and 60 min time points compared with the control group (Fig. 6). However, between 80 min and 100 min of heat stress, αB-crystallin levels in the H_9_C_2_ cells had recovered to normal levels compared to control cells.

## Discussion

Acute heat stress can be fatal due to sudden cardiac arrest or other heart disease [41]. Hyperthermia also has been shown to change the absolute number of cardiomyocytes in experimental animals [42]. Indicators for stress responses can be found by behavioral observations or by histological investigations [13], [43], [44]. In the present study, the SD rat model and H_9_C_2_ cell model were successfully established as research platforms for examining the effects of heat stress *in vivo* and *in vitro*, respectively. During heat stress, rats exhibited signs of nervousness, anxiety and thirst at the beginning (i.e., 20 to 40 min) of heat stress. After 60 min of heat stress, the rats had shortness of breath and increased drinking, and the mortality was 5% by this time point. With the extension of heat stress exposure time, the rats no longer drank and exhibited reduced physical activities. The mortality rate was 50% from 80 min to 100 min and 100% after 120 min of heat stress. Histopathologically, the degeneration of cardiac muscle fibers was accompanied by necrosis throughout the heat stress period (from 20 min to 100 min, Fig. 1b–f), while the H_9_C_2_ cells showed acute degeneration and nuclear pyknosis during 100 min of heat stress (Fig. 2b–f). These observations provide further confirmation that heat stress at 42°C can injure myocardial cells *in vivo* and *in vitro*.

Expression of HSPs has been considered to represent the response of cells to various stressors [18]. Their chaperone activities appear to be important in preventing damage and in cellular repair processes after injury [45]. HSPs from the HSP60 and HSP70 families have been widely investigated and are associated with heart vascular disease [12], [46]. Many studies also have reported that Hsp27, one of the sHSPs, can protect the heart [47], [48]. Although its specific function in the heart under heat stress has not been well-studied, αB-crystallin is known to have high chaperone activity [49]. HSPs are also present in cells under normal conditions by functioning as cytoskeletal proteins, which can stabilize myofilament proteins through selective interactions with actin, titin, nebulette and the intermediate filaments desmin and vimentin [50].

Our histopathological analyses, which were focused on variations of αB-crystallin and the relationship with pathological changes, demonstrated that heart tissue degeneration and disordered muscle fibers occurred as early as 20 min of heat stress (Fig. 1b–f). After 60 min of heat stress *in vivo*, the heart damage worsened and was accompanied by cell karyopyknosis and hyperemia (Fig. 1d–f). Meanwhile, expression levels of αB-crystallin were decreased until 100 min of heat stress, except at 40 min when it was significantly increased. This observation indicated that αB-crystallin initially was utilized heavily for its protective function to arrest the heat-induced unfolding of proteins [51]. During the course of the heat stress period, αB-crystallin may have become phosphorylated or converted into a soluble complex form to mediate reorganization of intermediate filaments in this pathological condition [52], [53]. After 60 min of heat stress, αB-crystallin levels in the rat heart decreased compared with the control group. Meanwhile, the mortality of heat-stressed rats increased sharply, reaching almost 50% after 100 min of exposure to high temperature. This observation suggested that there was an insufficient level of αB-crystallin *in vivo* to arrest the misfolding of proteins. Due to the high utilization of αB-crystallin which exceeded its production in myocardial cells at the beginning of the heat stress, the heat stress mediated induction of αB-crystallin was insufficient for cardiac protection when rats were subjected persistently to high temperatures. Furthermore, the immunohistochemical analysis of the rat heart showed consistently the localization of αB-crystallin in the cytoplasm and apparent decrease in density during heat stress compared to the control group. In particular, after exposure of the rats to a high temperature for 60 min, αB-crystallin was very weak in the cytoplasm of the cardiac cells, which was consistent with the Western blot analysis. The most severe pathological changes were accompanied by decreased expression of αB-crystallin, suggesting that the abundance of this protein is important to the response of rat hearts to heat stress *in vivo*.

However, different patterns of αB-crystallin were observed between the rat heart *in vivo* and H_9_C_2_ cells *in vitro*. In the H_9_C_2_ cells, the density of αB-crystallin increased after 80 min, and the strongest signal was observed at 100 min of heat stress. Interestingly, αB-crystallin signals were concentrated around and above the nuclei at the later time points, suggesting a dynamic shift in localization. αB-crystallin has been reported to interact with actin *in vitro*, and this interaction increases with increasing temperature [52]. Our preliminary *in vitro* results using the H_9_C_2_ myocardial cell line also showed that αB-crystallin levels were lower at 40 min and 60 min of heat stress compared with that of the control group. This decrease in αB-crystallin may indicate that it interacted with intermediate filaments to protect cytoskeletal organization in cardiomyocytes [53] in response to acute heat stress. A previous report indicated that heat shock can induce the redistribution and collapse of the intermediate filament networks, as well as cause αB-crystallin to interact with actin and desmin filaments *in vitro* [54].

While the density of αB-crystallin was observed in the H_9_C_2_ cells in this study to decrease between 40 min and 60 min of heat stress, cytopathological lesions manifested as acute cellular degeneration and necrosis (karyopyknosis, Fig. 2c–d). This phenomenon showed that the decreased levels of αB-crystallin were accompanied by severe damage to the myocardial cells *in vitro*. However, after 80 min of heat stress, expression levels of the αB-crystallin protein increased significantly (*P*<0.01), compared with those at 40 min and 60 min, and reached nearly the same level as the control.

When subjected to heat stress, proportions of αB-crystallin present in different soluble and insoluble forms can change, thereby altering the amount of total available αB-crystallin proteins in H_9_C_2_ cells [53]. In this study, cytopathological examination of the myocardial cell line showed an apparently low level of damage, with only acute degeneration but not necrosis, after 80 min of heat stress. In addition, the strongest positive αB-crystallin signals were observed in the cytoplasm, around and above the nucleus of H_9_C_2_ cells at 100 min of heat stress. Previous data have suggested that sHSPs can function as a protein chaperone in the nuclear compartment [55]. It is also possible for cytoplasmic chaperones to have related nuclear functions, such as importins which are both cytoplasmic chaperones for exposed basic domains as well as nuclear import receptors [56]. This explanation may account for the relative resistance of the H_9_C_2_ cells to the acute heat stress as demonstrated by the mild cellular damage, although the potential mechanism for the transport of sHSPs into the nucleus is not fully understood. The above findings were also in line with the Western blot results, suggesting that the variation in expression of αB-crystallin are related to cell damage and that this protein may play a protective role in heat-stressed H_9_C_2_ cells. However, the specific localization and function of αB-crystallin in cardiomyocytes *in vitro* remain to be investigated in further studies.

Despite indications that αB-crystallin expression is a protective response, the reason for the high mortality rate in heat-stressed rats remains unclear. As mammals, rats can radiate heat through sweat glands while being subjected to heat stress at 42°C. By contrast, broiler chickens are more sensitive to high ambient temperatures due to a higher body temperature, rapid metabolism and the absence of sweat glands [57]. In the current study, αB-crystallin was expressed at lower levels in rat hearts with more severe tissue damage after 100 min of heat stress, compared with those after 60 min of heat stress. However, in the H_9_C_2_ cell line, the pathological changes were mild after 80 min of heat stress, which was different from the changes seen *in vivo*. From the analysis by immunofluorescent staining, αB-crystallin was observed to be localized in the cytoplasm of rat cardiac cells before and after heat stress *in vivo*. However, αB-crystallin localized not only to the cytoplasm, but seemed to migrate or accumulate towards the nucleus in H_9_C_2_ cells after 60 min of heat stress *in vitro*. Previous *in vitro* studies have confirmed that αB-crystallin is mainly present in the cytoplasm, but it also has roles in splicing [58] and protection against apoptosis in the nucleus [59]. Therefore, αB-crystallin seems to function both in the cytoplasm and in the nucleus.

There may be yet other reasons for the differences in the degree of damage observed *in vivo* and *in vitro* in our study. Recently, αB-crystallin was reported to form polydisperse hetero-oligomers *in vitro* during heat stress, which had an average molecular mass that was intermediate between each of the homo-oligomers and which were more thermostable [60]. However, whether αB-crystallin functions in such a manner in the heart to protect cells heat-stressed cardiac cells and reduce mortality is an open question. Furthermore, αB-crystallin was expressed at a very high level in dead rats compared to other groups, which was interesting but inexplicable in this study. Previous studies have concluded that overexpression of HSPs may increase vulnerability of mature cardiac myocytes or cause other unexpected effects [61], [62]. While the above interpretations are speculative, the present study showed that expression patterns of αB-crystallin in heat-stressed rat hearts and H_9_C_2_ myocardial cells were consistent with the observed pathological changes. Thus, our results demonstrated that αB-crystallin may play a protective role in cardiac cells subjected to heat stress *in vivo* and *in vitro*. However, our comparative analysis suggests that the mechanism of this protection is not the same between the individual cell and the whole body, and it may be related to the form and subcellular location of αB-crystallin when subjected to heat stress. These findings prompt further in-depth investigations into the specific mechanism of αB-crystallin-mediated protection in the heart.
